# Digital Dermatitis in Cattle: Current Bacterial and Immunological Findings

**DOI:** 10.3390/ani5040400

**Published:** 2015-11-11

**Authors:** Jennifer H. Wilson-Welder, David P. Alt, Jarlath E. Nally

**Affiliations:** Infectious Bacterial Diseases of Livestock Research Unit, National Animal Disease Center, Agricultural Research Service, United States Department of Agriculture, Ames, IA 50010, USA; E-Mails: david.alt@ars.usda.gov (D.P.A.); jarlath.nally@ars.usda.gov (J.E.N.)

**Keywords:** digital dermatitis, treponemes, anaerobes, immune response, dairy cattle, J0101

## Abstract

**Simple Summary:**

Digital dermatitis causes lameness in cattle. Numerous studies have identified multiple bacteria associated with these painful lesions. Several types of a spiral shaped bacteria, *Treponema* species, are thought to play a role in disease development. Little is known about the immune response to bacteria involved in digital dermatitis. Local inflammatory cells can contribute to the non-healing nature of the disease. Animal models of infection are required to develop effective vaccines and treatments.

**Abstract:**

Globally; digital dermatitis is a leading form of lameness observed in production dairy cattle. While the precise etiology remains to be determined; the disease is clearly associated with infection by numerous species of treponemes; in addition to other anaerobic bacteria. The goal of this review article is to provide an overview of the current literature; focusing on discussion of the polybacterial nature of the digital dermatitis disease complex and host immune response. Several phylotypes of treponemes have been identified; some of which correlate with location in the lesion and some with stages of lesion development. Local innate immune responses may contribute to the proliferative, inflammatory conditions that perpetuate digital dermatitis lesions. While serum antibody is produced to bacterial antigens in the lesions, little is known about cellular-based immunity. Studies are still required to delineate the pathogenic traits of treponemes associated with digital dermatitis; and other host factors that mediate pathology and protection of digital dermatitis lesions.

## 1. Introduction and Digital Dermatitis Lesion Descriptions

Lameness is the second largest issue affecting dairy cattle health [1] and poses a serious economic burden on producers due to lost production, increased reproductive intervals, increased culling, and cost associated with footbaths and treatment. Furthermore, lameness and animal welfare are interconnected. Changing public perception and increased focus on how food is raised has placed pressures on animal agriculture which are reflected in both regulatory approaches and in consumer driven willingness to pay for products from high-welfare farms [2]. On farm studies have observed that lameness can range from 5% to 37% of animals in the milking population [3,4,5]. Depending on geographic region, data suggests that 10%–40% of all lameness cases can be attributed specifically to digital dermatitis (DD) [6]. The earliest reports of DD, commonly called hairy heel wart, strawberry heel, or raspberry warts, were from dairy herds presenting with severe lameness. Individual animals showed decreased mobility, lifting of the affected leg or walking with a toe down posture [7,8,9,10]. The disease has now been described throughout much of the world in high density housing and intensive production dairy systems. Other reviews appearing in this special issue and recently published elsewhere highlight herd and individual risk factors for DD [11,12]. This review gives a brief introduction to DD lesion descriptions, followed by current knowledge of the bacterial pathogens associated with DD and host immune response to DD.

### 1.1. DD Lesion Description

**Figure 1:**
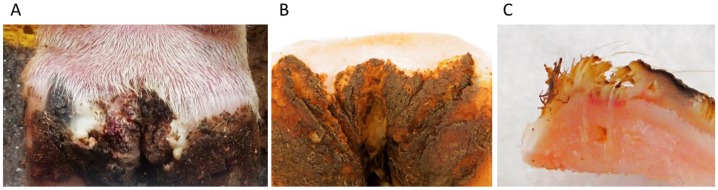
Bovine Digital Dermatitis. (**A**) A characteristic bovine digital dermatitis lesion on the left rear foot of a female adult Holstein cow; (**B**) M4.1 digital dermatitis lesion on the rear foot of a female adult Holstein cow; (**C**) Cross-section of the inactive lesion in (**B**), showing a central area of active hyperemia and congestion under the crust-like scab. This lesion was positive for the presence of spirochetes. Images generated from author’s research, previously unpublished.

A typical active lesion associated with bovine DD as shown in Figure 1, is found on the plantar surface of the hind foot of a dairy cow which presents as a circumscribed moist ulcerative erosive mass along the coronary band or interdigital space [13]. Lesions initially present as small (1 cm) flat to raised erythematous masses with papilliform projections. Histologically, there is a loss of stratum corneum and/or granulosum, invasion of stratum spinosum by spirochetes, epidermal hyperplasia, and reactive inflammation (infiltration of neutrophils, plasma cells, lymphocytes, and eosinophils in dermis) [13,14]. Over time, lesions can become larger, develop frond-like projections and are prone to ulceration or physical trauma. Pain upon palpation and lameness is often but not always present; lesions are prone to bleeding when touched [15]. Although most often seen in dairy cattle, DD also occurs in beef cattle [16,17,18]. Recently, what best can be described as DD-like disease based on histopathology and bacterial involvement has also been observed in sheep, goats, and wild elk (reviewed in [11]) [18,19,20,21,22]. Although these lesions present different clinically, involving the coronary band and underrunning the hoof capsule, it is apparent that treponemes are a major pathogenic complex detected in nearly all lesions. Similar bacterial involvement, histologic pathology and treatment has led some researchers to consider DD as a spectrum of clinical lesions in cattle and other ruminants including interdigital dermatitis [23]. Consideration of DD as part of a spectrum of hoof diseases has also been proposed with detection of DD associated bacteria in other non-healing hoof conditions (*i.e.*, “non-healing” sole ulcer, toe necrosis, white line disease) [24].

Efforts to describe or classify DD lesions have resulted in several different scoring systems. Most describe the lesions in an early ulcerative or granulomatous phase (Figure 1A) passing through to a dyskeratosis and proliferative phase, developing into a chronic or persisting lesion (Figure 1B). It is important to note that one animal may have lesions in multiple stages and even within a lesion there may be areas of both chronic proliferation and active hyperemic ulceration (Figure 1C) [25,26]. More detail on lesion scoring and progression of lesion development can be found in another recent review [11].

### 1.2. Multiple Treponema Associated with DD

DD is an infectious disease; the rapid spread after introduction of new animals into a herd consistently supports this hypothesis [27]. Although no definitive etiologic agent has been identified, numerous targeted and genome-wide shotgun sequencing studies have consistently indicated that viral and fungal pathogens are not associated with DD [9,28,29]. DD is a polybacterial disease complex as evidenced by the multiple different bacterial agents that have been cultured and identified from active DD lesions [26,29,30,31,32,33,34,35,36,37,38,39,40,41,42,43]. This is further supported by the improvement or resolution of clinical lesions in response to antibiotics [25,44,45,46,47,48,49,50,51,52,53,54]. The most common bacteria associated with DD include multiple species from the genus *Treponema*.

Determination of treponeme types or species associated with DD lesions has been based on DNA sequence analysis and classification. Evans *et al.*, established the three most common phylotypes, *T. vincentii/T. medium*-like, *T. phagedenis*-like and *T. denticola/T. putidum*-like, clustered on 16S rDNA homology and *flaB2* homology [55]. Phylotypes (PT) are defined as clusters of treponemes in which the 16S rDNA sequence differs by ~2% from known species and which are ≥99% similar to other members of their cluster [36]. Others have expanded the number of phylotypes up to seven including *T. brennaborense*, *T. maltophilum*-like (including *T. maltophilum* and *T. lecithinolyticum*), *T. refringens/T. calligyrum*-like, and *Spirocheta zuelzerae*, with *T. pedis* clustering with *T. denticola/T. putidum* [23,36,54,56,57]. Within these clusters or phylotypes, there are over 17 genomospecies, where the 16S rDNA homology is 98% or greater [57]. A small number of California isolates were typed by 16S–23S rDNA intergenic spacer regions, and the isolates were grouped into similar clusters [58]. The treponemes associated with DD are not the same as those found in the rumen, forming distinct clusters by 16S rDNA sequence analysis [59]. Evidence suggests that treponemes identified from DD lesions around the globe are similar by 16S rDNA.

Different studies have provided varying results as to the dominant phylotypes present. Nordhoff *et al.*, detected *T. phagedenis*-like group, TRE I (*T. vincentii*-like), TRE IV, TRE II (*T. denticola*-like) and DDKL-12 in 100%, 83%, 82%, 80%, and 66% of the samples, respectively [60]. *T.* *phagedenis*-like and *T. vincentii*-like phylotypes were found at the interface of healthy and affected tissues. Brandt *et al.*, observed in the DD samples included in their study: *T. pedis*-like treponemes (by specific PCR probes), *T. medium*-like isolates, TRE IV treponemes, and a phylotype previously not identifed in 51%, 30%, 16% and 11% respectively [31]. A recent study in a closed bovine herd identified a large number of sequences from the genus *Treponema*, containing 45 unique species, with 12 species being the most predominant [29].

Prevalence of the different phylotypes differs according to stage of lesion development as well as the location within the lesion. Identification of multiple phylotypes of treponemes by *in situ* hybridization indicate both *T. phagedenis* and *T. vincentii* types appear to be highly invasive with *T. refringens*-like and PT3 (*T. calligyrum*-like) located more superficially [35,57,61]. Krull *et al.*, demonstrated that different phylotypes dominate the lesion at different stages of development [29]. While *T. phagedenis* was present at all lesion stages (early, erosive, proliferative, chronic, and healed) treponemes dominating the early lesions most resembled uncultured, unidentified *T. refringens*-like PT1, PT2, PT3 (*T. calligyrum*-like) [29]. In mature or chronic lesions, a novel *T. refringens*-like, *T. medium*, *T. pedis*/PT8, and *T. denticola* were the most common treponeme operational taxonomic unit (OTU)s identified. It is interesting to note while *Treponema* were the most numerous phyla in the mature and chronic lesions, in the early stages, treponemes were less than 15% of the total OTUs [29]. Adding to the difficulties in interpretation of these findings is the observation that not every study identifies every phlyotype. Despite the use of *T. brennaborense* specific oligonucleotide probes in multiple studies, *T.* *brennaborense* was not always detected [35,62], suggesting that there may be regional/geographical variance in DD-associated treponemes.

**Figure 2:**
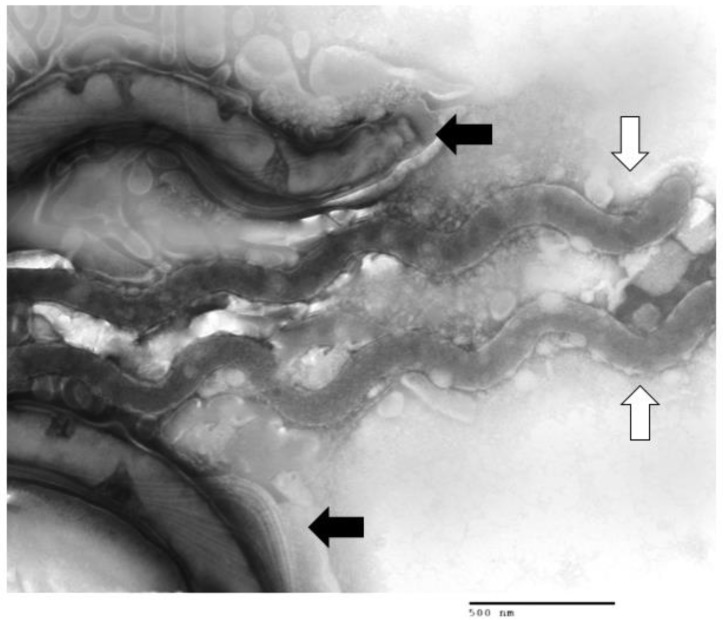
Transmission Electron Micrograph of Multiple Treponeme Morphologies Isolated from DD lesion. TEM of broth culture inoculated with DD lesion tissue homogenate showing multiple Treponeme morphologies: black arrows indicate one morphotype, white arrows indicate a second in the same sample as determined by flagella numbers (not visible), full length and width. Image generated from author’s research, previously unpublished.

*T. phagedenis* (or *T. phagedenis*-like) are the most readily isolated treponemes from bovine lesions [63,64,65]. Treponemes cultured from DD lesions collected in various areas of Japan yielded mostly isolates of *T. phagedenis*-like and a few *T. denticola*-like treponemes [65]. Difficulty in obtaining other isolates may result from the strict anaerobic conditions required to maintain growth after initial isolation or lack of nutritional or co-dependent requirements [66]. Figure 2 illustrates multiple treponeme morphologies co-isolated from a DD lesion. With similar growth requirements, separation of two co-isolated spirochetes can be difficult.

Analysis of bacterial 16S rDNA isolated from DD lesions only reveals the level of diversity. This analysis is narrow, by its nature, limited, and does not capture the full genomes of these treponemes or depict the potential functional diversity of their full genomes. Genomic comparisons of DD-associated *Treponema* have been limited to a few studies using *flaB2* sequences, pulse-field gel electrophoresis (PFGE), random amplified polymorphic DNA (RAPD), and functional comparisons mainly consisting of enzymatic activity as measured by commercially available kits (apiZYM) [55,63,65,67,68,69,70,71]. For most DD isolates, little direct work has been done on virulence attributes.

Many members of the *Treponema* genus are associated with polymicrobial periodontal disease of humans and companion animals, possessing a large number of classical virulence attributes such as adhesins, hemolysins, (host) protease modulators, immune evasion mechanisms, nutrient transporters, proteases, and motility [72]. Another example of treponeme involvement directly in chronic ulcerative or proliferative dermatosis is *T. pedis*. *T. pedis*, while also associated with DD, has also been implicated in porcine skin ulcers [73,74,75], cankers in horses [76,77,78,79], and a related treponeme is isolated from perioral and genital chronic ulcerations in European wild hares [80]. Virulence attributes present in DD-associated *Treponema* based on their involvement in other diseases, could indicate their role in bovine DD lesion development and perpetuation.

Treponemes have also been implicated in a number of other chronic infections in cattle beyond DD. Recently the presence of DD *Treponema* sp. has been observed in association with other forms of lameness including toe necrosis, sole-ulcer, and white line disease. Interestingly, these were all characterized clinically as non-healing, suggesting the potential for colonization of physically compromised hoof tissues by treponemes [24]. Bovine ulcerative mammary dermatitis has also been associated with *Treponema* sp. genetically similar to those found in DD [81,82]. The presence of treponemes in bovine interdigital cuts or wounds indicates their abundance in the production environment and their potential to colonize/invade damaged skin [83].These sites represent regions beyond those normally associated with DD lesions. The authors proposed that *Treponema* organisms, present in DD endemically affected farms, play a role exacerbating other hoof diseases, and contribute to the development of the non-healing state [33,81,82]. The fact that similar organisms have been observed in multiple anatomic sites and on different species (sheep, swine, horses, and cattle) and in unrelated hoof diseases, speaks to the opportunistic behavior of *Treponema* for affecting compromised tissue [17,73,74,76,77,78,79,84,85]. The presence of treponemes in a collection of chronic ulcerative dermatoses suggests the presence of common virulence attributes that may include metabolic pathways, mobility, and persistence in the environment, which synergistically exacerbate clinical symptoms/lesions.

In human periodontal disease, another chronic treponeme-driven lesion, the development of molecular detection tools and ease of metagenomic sequencing has greatly expanded knowledge of these multifactorial lesions in recent years. Application of molecular detection tools has shown a greater diversity of bacterial organisms than was previously determined by culture methods alone [86]. It is estimated that in human periodontal disease, 70% of *Treponema* species remain uncultivable [87]. Molecular methodologies including PCR, genomic sequencing, and other DNA based methods have helped elucidate bacterial members in periodontal disease, but without cultivable isolates, insight into interplay of the bacterial community has been slow [88]. Similar studies into bovine DD focused on molecular detection have identified a number of previously uncultured *Treponema* from DD lesions [26,29,35,36,65]; this would suggest that like periodontal disease, DD involves a similarly large number of uncultivated and unidentified bacteria.

Historically, proteases of *Porphyromonas* (*Bacteroides*) and other bacteria were considered the main cause of tissue necrosis in human periodontal disease; and that treponemes were secondary invaders. However, many small oral treponemes (including *T. vincentii* and *T. denticola*) and the non-oral non-pathogen *T. phagedenis* have potential for tissue degrading enzymatic activity [71,89]. *Treponema* (*T. denticola*, *T. vincentii*, and *T. medium*), isolated from both sheep and cattle, bound to fibrinogen and fibronectin and co-aggregated with periodontal pathogens *Porphyromonas gingivalis*, *Streptococcus crista*, *Fusobacterium nucleatum* and *F. necrophorum* [90]. Other putative virulence factors of several treponemes (representing phylotypes 1, 2, and 3) include homologous genes to known hemolysins [59]. Analysis of several *T. vincentii*, *T. denticola*, and *T. phagedenis*-like isolates from both sheep and bovine DD indicate they possess chymotrypsin-like proteases, trypsin-like protease, proline iminopeptidase, and demonstrate esterase activity [55,90]. Enzymatic activity by one or more treponeme phylotypes possibly contributes to tissue destruction observed on histological evaluation. *T. pedis*, isolated from DD lesions of cattle, shares many virulence factors with *T. vincentii*, *T. denticola*, and *T. phagedenis*, including C4 and C8 esterase, serum dependence, trypsin, and chymotrypsin activity [69]. Comparative analysis of *T. pedis* to *T. denticola* genomes revealed similarities in virulence factors including several proteases, hemolysins, and a surface antigen involved in co-aggregation with *Tannerella forsythia* [75]. While similarities exist with dental or other treponemes, comparing isolates from widely differing ecological niches may diminish the unique attributes of the DD treponemes. Whole genome comparison also revealed that *T. pedis* contained more energy-production genes than *T. denticola*, possibly a consequence of a wider host and niche (skin of ear, shoulder, and hoof and oral cavity) range in *T. pedis* [75].

Like *T. phagedenis*, *T. refringens*, and *T. calligyrum* are categorized as non-pathogenic commensals of human and animal genitalia [91]. Experiments with *T. phagedenis* isolates have shown inhibition of innate immune responses in a bovine macrophage-like cell line, and abscess formation in mice [92,93]. Finally, the suggestion that DD-associated treponemes can persist in encysted forms, much like *T. pallidum* or *Borrelia* sp., has implications for chronicity of lesions, evasion of immunity, reoccurrence, and environmental persistence [68]. Detailed analysis of type strain of *T. phagedenis* biovar Kazan and *T. phagedenis*-like isolates from Iowa showed that they had a high degree of similarity in DNA-DNA hybridization, nearly identical enzyme activity profiles, the same growth tolerances and same number of flagella, indicating these *T. phagedenis* isolates are the same species, obtained from different hosts and anatomic locations [71]. Further analysis is needed to find if there are unique functions or virulence attributes of the hoof-associated *T. phagedenis* isolates to distinguish them from others. Likewise, studies need to continue isolating and characterizing other DD-associated treponemeal isolates. Looking at genes beyond 16s rDNA may show that previously clustered 16S rDNA phylotypes do contain unique genes or functions associated with life on the bovine foot. By identifying similarities and differences in *Treponema* associated with DD lesions, work can begin toward targeted therapeutics and interventions.

### 1.3. Other Bacteria Associated with DD

**Figure 3:**
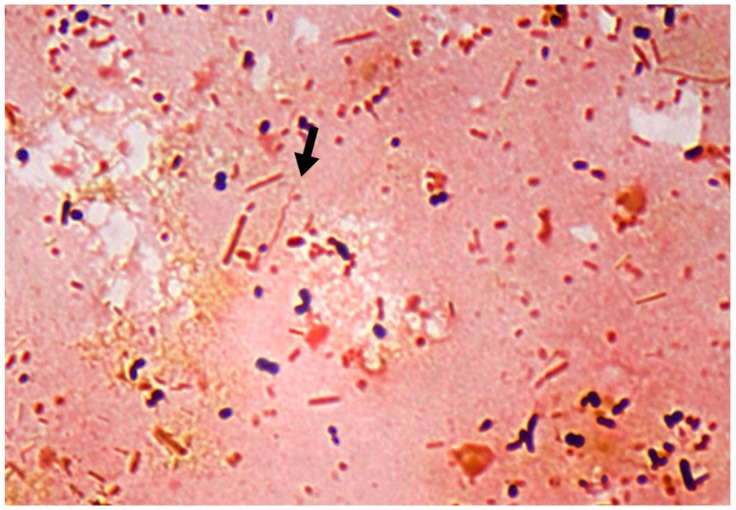
Gram Stain of DD lesion tissue homogenate. A characteristic Gram Stain with phenol-red counterstain of tissue homogenate from a DD lesion showing multiple bacterial shapes including Gram+ cocci (purple), Gram—rods (red), and Spirochete (arrow). Image generated from author’s research, previously unpublished.

While treponemes are closely associated with DD lesions, it is theorized that a number of other bacteria are required to facilitate skin colonization, lesion development, and chronicity. The Gram Stain in Figure 3 demonstrates multiple bacterial morphologies associated with a DD lesion. Further evidence for involvement of other anaerobes includes the observation that antibody responses in cattle with active or recent DD have higher levels of reactive IgG to antigens from *Porphyromonas*, *Fusobacterium*, and *Dichelobacter* than cattle without lesions [23,38].

In an early study of bovine DD lesions, anaerobic bacteria *Peptostreptococcus*, *Peptococcus*, *Bacteroides*, *Fusobacterium*, *Streptococcus*, and *Clostridium* were all isolated from DD tissues, with *Bacteroides* and *Fusobacterium* found in over 50% of the samples [94]. A study of the microbial diversity in DD lesions from dairy cattle in upstate New York showed that superficially, *Firmicutes* were the most significant and diverse phyla associated with superficial and intermediate zones of the lesion, where *Treponema* dominated the deep layers of the lesion [95]. This same study also detected a number of archaea, the first and only one to do so [95]. Sequencing of a number of European samples by 16S rDNA showed that while 50% of the sequences were *Treponema*-like, 25% were of *Fusobacterium necrophorum*, and the remaining were similar to *Streptococcus dysgalactiae*, *Pasteurella* sp., and *Klebsiella oxytoca* [35]. A Brazilian study of diseased hoof samples from slaughterhouses and dairy farms showed that of 159 total samples, 111 had visual presence of spirochetes, 144 rod-like bacterial forms, 91 coccoid structures, and 61 had filamentous branching forms, with many lesions having multiple types (spirochetes, rods, and coccoid) present [33]. Using antibodies to detect *Campylobacter* and *Fusobacterium*, these two bacterial genera were associated with a large number of DD and interdigital dermatitis samples [83]. Filamentous, branching forms were morphologically consistent with *Actinomycetes*, a common pathogen in human periodontal disease, and non-healing wounds [83]. *Fusobacterium necrophorum* and *Porphyromonas levii* antigens were detected in DD lesion biopsies in Japan by western blot [38]. Frequently isolated along with treponemes, are black pigmented bacteria, some of which have been identified as *Porphyromonas levii*, which are also associated with the pathogenic complex of bacteria in periodontal disease [38] (Wilson-Welder, unpublished observations). Other sequencing studies in the UK have found a number of sequences from DD lesions that correspond to *Porphyromonas* (*Bacteroides*) *levii* and *Mycoplasma hyopharyngis* [32]. Krull *et al.*, indicated that the relative abundance of *Mycoplasmataceae*, *Moraxellaceae*, and *Porphyromonadaceae* were higher in early DD lesions than healthy tissue samples [29]. A number of researchers at different times and geographic locations have isolated unique *Campylobacter* species from DD lesions [34,39,96]. These studies did not identify these anaerobic bacteria as either primary or secondary colonizers in DD lesions, which may be important in disease development. *Campylobacter*, *Fusobacterium*, and *Bacteroides* species of varying types are also known to colonize/invade compromised epithelial tissue [39,83].

Two other bacteria of interest that have been isolated and associated with DD lesions are *Guggenheimella* species and *Dichelobacter* *nodosus*. Isolates of *Guggenheimella* from DD lesions had C4 and C8 esterase, chymotrypsin activity, and produced β-hemolytic colonies on anaerobic Columbia blood agar plates [43]. Much like the treponemes, *Guggenheimella* probes showed these organisms deep within DD lesions, and not in the superficial bacterial biofilm covering DD lesions [40]. Two different analyses of microbial diversity in DD lesions in the US and Japan identified the phyla/family *Tissierellaceae* in early stage lesions, but the resolution of genomic OTUs presented was not sufficient to determine if *Guggenheimella* species were present [29,61]. The role or prevalence of *Guggenheimella* in DD is still unclear.

*Dichelobacter nodosus* in conjunction with *Fusobacterium necrophorum* is globally recognized as the causative agent in foot rot of sheep and goats. *D. nodosus* was detected in a number of DD lesions from different geographic locations [23,57,84,97]. The finding of *D. nodosus* in DD lesions from dairy cattle in the U.S., where comingling of cattle and sheep on pasture is uncommon, suggests that *D. nodosus* has a role in the pathogenesis of DD, and is not a secondary invader or present merely because it is ubiquitous in the animal’s environment [29]. *D. nodosus* produces extracellular proteases assumed to be associated with tissue damage and can be readily co-detected with treponemes in interdigital dermatitis and heel horn erosion lesions. Thus, *D. nodosus* is hypothesized to act in synergy with treponemes to initiate DD [23,57,98,99]. *D. nodosus* is notoriously hard to culture, with only a few specialized labs having success [84,100,101]. PCR based detection strategies are often employed for detection of *D. nodosus*, with specific primers that allow for differentiation of virulent and benign strains [98].

Overall, data from numerous studies using multiple methodologies (sequencing, direct culture, immune-detection, fluorescent *in situ* hybridization, and host response) indicate that DD is a polymicrobial disease. The confusion and debate as to which “other” bacteria are involved continues, as different studies separated by time, methods, and geography, have yielded different results. As is the case with periodontal disease, there may be a multitude of bacterial species involved in bovine DD. There may not be a single (or even multiple) bacterial species that is always present in lesions, but instead a number of interchangeable species with a core set of virulence factors or metabolic pathways that create a favorable microenvironment for treponeme invasion, cause alteration of host response, or other means of lesion perturbation. In studying the microbial makeup of DD lesions, it would be short sighted to evaluate only the *Treponema* sp. and not include analysis of “other” anaerobic bacteria isolated from affected tissues. Evaluation of the oral microbiome in chronic periodontitis has shown that there is considerable variation in the bacterial species present from one patient to another, and even in one dental pocket to another in the same patient [102,103,104]; but when the functional signatures of the bacteria were compared, a high degree of correlation between disease, resolution, and ultimately patient prognosis was seen [103,104]. Zincola *et al.*, published a functional composition analysis of the bacterial metagenome comparing active and inactive DD to healthy skin communities. Much like the chronic periodontitis-associated samples, DD lesion samples had an abundance of genes associated with bacterial motility/chemotaxis (flagella), iron metabolism, phosphorus metabolism, and metabolism of aromatic compounds. Interestingly, genes associated with antibiotic resistance, multidrug efflux pumps, copper homeostasis/tolerance, and cobalt-zinc-cadmium resistance present in higher abundance in DD lesion samples [105]. This indicates that some members of the microbial community may be able to resist the effects of footbath or other topical treatments for DD as these commonly contain antibiotics, zinc or copper compounds. As more studies of this type become available, comparisons and inferences about a functional metabolic signature for DD can be made. Little is known about the early colonizing and initiating events of DD. By studying the bacterial functional signatures of healthy skin, early and chronic lesions, researchers may gain insight into bacterial community development and disease progression which may lead to improved diagnostics or therapeutics.

## 2. Bovine Immune Response to DD

Treponemes associated with DD have been shown to induce limited humoral and cell-mediated immune responses. Serum antibody reacts with high affinity to antigens derived from treponemes [64,106,107,108,109]. There is a wide range of magnitude or level of serum antibody response from individuals within groups containing animals with active lesions, recovered lesions, and presumed naïve groupings [108,110] (Wilson-Welder, unpublished data). Variability in immune responses may be partially explained by different phylotypes of treponemes found in DD lesions and mismatch to antigens used in assays. Furthermore, it is hypothesized that treponeme and bacterial populations shift over time [29], are spatially distributed within the lesion, and thus provide little or limited contact with the host immune system. Non-pathogenic treponemes are part of the normal intestinal flora; their presence could lead to immunologic tolerance and a lack of an antibody response [96,111]. Overall, differences in host reactivity, number of potential antigens and pre-existing responses makes serology of limited usefulness, since paired sera from naïve and affected animals are needed to compare changes in response.

Information on cell-mediated immune responses to DD-associated bacteria is limited. Studies using a bovine macrophage cell line incubated with *T. phagedenis* isolated from BDD (Iowa strain 1A) showed increased expression of genes regulated by NF-κB and other cell signaling associated molecules, increased expression of apoptosis associated molecules (BCL-2), down-regulation of immune modulation pathways, antigen presentation and cytoskeletal rearrangement, and wound healing pathways [93]. This represents a single cell type interacting with a whole cell sonicate of a single bacterium present in the DD lesion; it provides a small snapshot of the complexity of host-pathogen cross talk. In humans, peripheral blood mononuclear cells (PBMCs) stimulated *in vitro* with *T. denticola* antigens produced IFN-γ and IL-17, two cytokines associated with adaptive immune responses. Cytokine production was impaired in PBMCs from patients with chronic periodontal disease, indicating a bias for, and protective role for cell-mediated, rather than humoral-biased adaptive immune responses [112]. PBMCs from infected cattle proliferated when incubated with treponemal antigen, a large percentage of which were γδ-T cells [64]. In ruminants, γδ-T cells comprise a large number of the circulating lymphocytes, 15–60% depending on age [113]. These T cells can have both innate-like functions and antigen specific adaptive like functions, and may even act as suppressive, regulatory T cells [114]. As with human periodontal disease, cell-mediated immune responses may be more protective in DD and more informative in diagnostic assays. However, this is an area that needs further study.

Analysis of total RNA transcripts in DD lesions and normal skin indicated no activation or suppression of the local immune response [115]. Matrix metalloproteinase (MMP)-13, a cytokine secreted by many cell types involved in tissue remodeling, was increased in DD lesions [115]. However, DD lesions had downregulated expression of genes encoding keratin and keratin-associated proteins [115]. In another study, whole cell sonicates of treponemes induced innate immune inflammatory responses in bovine foot skin-derived fibroblasts, including cytokines RANTES/CCL5, MMP-12, TNF-α, TGF-β, and TIMP3. In comparison, no significant changes were observed using bovine foot derived keratinocytes [116]. The authors concluded that fibroblasts, not keratinocytes, were responsive to *Treponema* co-culture and contributors to inflammation in DD lesions [116]. Indeed, keratinocytes are mainly limited during infection to proliferation (e.g., removal of the pathogen by sloughing the area) and production of cytokines to recruit inflammatory cells (*i.e.*, neutrophils). Upon entering the area, neutrophils encountering pathogens secrete more cytokines and chemokines which enhance tissue regeneration and recruit more inflammatory or immune mediating cells such as macrophages and plasma cells [117]. Thus keratinocytes and neutrophils create a feedback loop that perpetuates lesion growth and inflammatory conditions as long as treponemes or other bacteria remain present.

In addition to circulating lymphocytes, tissue resident lymphocytes have recently been highlighted as being important in protection from disease. The skin harbors a large number of resident memory T cells (TRM) which can respond to antigen and drive local immune responses, both in allergy, hypersensitivity, and protection from pathogens [118,119,120,121]. These TRM cells have particular homing signals consisting of surface ligands (CCR7, CCR8, CCR4, and CD69) [120,121] triggered by costimulatory signals from innate immune cells and possibly the presence of vitamin D3 metabolites [119]. Small lymphocytes have been observed in or adjacent to DD lesions [34,64], but little has been done to characterize these cells or elucidate their role in lesion development or immunity. As cell-mediated adaptive immune responses are the goal of most successful vaccines, it is necessary to understand the host/bovine immune response induced by natural infection in order to find the best ways to enhance or overcome existing responses.

## 3. Disease Model and Further Research Needs

Bovine DD has been experimentally reproduced using homogenized lesion material [122]. This model included lengthy preparations of wet wraps creating an anaerobic, compromised environment. Attempts to induce DD lesions with pure cultures of *T. phagedenis* have been unsuccessful and use of a clonal isolate of a *T. vincentii*-like organism was only marginally successful [63,122] (Alt, unpublished data). A recent study failed to observe transmission from clinically affected cows co-housed with eight healthy heifers over a period of eight weeks despite housing and environmental modifications in an attempt to enhance transmission [62]. This contradicts field observations of frequent lesion development after introduction of new animals into an affected herd. This highlights the hypothesis that DD is not just a polytreponemal and polymicrobial disease, but suggests there are other complicating factors that can be complex and variable. Another review in this issue details many of these factors [12]. Predisposing factors such as immunosuppression, negative energy balance in early lactation or poor hoof cleanliness are difficult to replicate in a research setting making model development anything but straightforward. In experimental models of disease, the native host is always best, however close substitutes may be more practical. Mature bovines present considerable logistic challenges for evaluation of hooves on a daily basis without specialized equipment. Other small ruminants (sheep or goats) have proven susceptible to DD-like disease, and may be a feasible alternative. A mouse abscess induction model, commonly used in periodontal disease research, has been used to evaluate pathogenesis of *T. phagedenis* DD isolates [92]. As no animal model captures all aspects of human periodontal disease, no single animal model may perfectly replicate DD outside of the bovine host. However, laboratory animal models can provide insight on bacterial invasion, bacterial interactions, host responses, or other specific hypothesis driven questions within a complex cellular system involving epithelial, immune, and repair components that cannot be replicated in cell culture systems [123].

## 4. Conclusions

While current measures to combat DD limit the on-farm impact of disease [11,44,53,124,125,126,127,128,129,130,131,132], these are not without risks. Antibiotics are under close scrutiny and face ever tightening restrictions for use in food-producing animals [46,133,134]. Formalin and copper sulfate used in footbath solutions have potential environmental and human health risks [135,136,137,138]. Research efforts to develop effective vaccines or other targeted therapeutics for DD need to continue. DD is a multifactorial, multibacterial, and multi-treponemal disease. Local innate immunity may exasperate and perpetuate the lesion in the continued presence of the bacteria. The role of systemic or adaptive immune response is largely uncharacterized. Thus, reproducible animal models need to be developed that allow researchers to understand the identity of the bacteria in the lesions, interactions with each-other, and the host. How the lesion is created and perpetuates and will allow for hypothesis driven investigations into immune-mediated protection. Efforts to isolate and culture the bacteria involved in the lesions, especially treponemes, need to continue. Virulence traits, and appropriate intervention strategies, can only be identified if individual isolates are evaluated. While much can be surmised from similar disease processes in periodontal disease, evidence would indicate that the bovine hoof and its environment pose unique challenges to the pathogenic consortium in DD. While much has been learned about DD in recent years, there is still a long way to go in complete understanding.
